# Confidence, skills and barriers to ostomy patient care by nursing staff in Saudi Arabia

**DOI:** 10.1111/nuf.12701

**Published:** 2022-02-01

**Authors:** Aishah Alenezi, Amanda Kimpton, Ian McGrath, Karen Livesay

**Affiliations:** ^1^ Applied Health Department Discipline of Nursing, RMIT University Melbourne Victoria Australia; ^2^ Chiropractic and Exercise Sciences Department RMIT University, Victoria Melbourne Australia

**Keywords:** nurse confidence, ostomy care, ostomy patient

## Abstract

**Aim:**

This study examined the confidence level and perceived barriers to providing ostomy care among staff nurses in Saudi Arabia.

**Background:**

Patients with ostomies experience increased comfort and satisfaction when nurses are confident in their knowledge and skills.

**Design:**

A descriptive, cross‐sectional design was used to conduct the research. The study included a convenience sample of 214 staff nurses from five hospitals in Riyadh, Saudi Arabia. The researchers used a survey questionnaire to gauge participants’ confidence in their knowledge and skills, as well as identify any perceived barriers to ostomy care.

**Result:**

Overall, 95.1% of participants worked in adult care and 82.2% worked in surgical areas. There were significant relationships between the nurses’ confidence in their ostomy care knowledge and skills and their years of nursing experience and having received ostomy care training in nursing school (*p* < .05); however, the nurses’ level of education had no correlation (*p* > .05).

**Conclusion:**

While the majority of nurses were confident in their ability to care for patients with ostomies, they were concerned about causing stoma problems. This suggests that improving the quality of ostomy care provided by nurses may result in fewer negative outcomes for patients with ostomies.

## 1 INTRODUCTION

Globally, colorectal cancer is the third most commonly diagnosed cancer in men and the second in women, with the highest incidence rates in North America, Europe, Japan, Australia, and New Zealand. In Saudi Arabia, colorectal cancer is the most commonly diagnosed cancer which considered as the first diagnosed cancer among men (10.6%) and the third in women (8.9%).^1^ An ostomy is a surgical procedure in which the bowel or ureter is attached to the abdominal wall to create an opening (stoma) for feces or urine elimination.^2^ Ostomies are difficult procedures that can have a major impact on an individual's physical, psychological, social, and spiritual well‐being.^3^, ^4^ Some of the associated lifestyle changes include the need for daily care of the ostomy and peristomal skin, properly fitted equipment, appropriate diet and elimination strategies, flatus and odor control, and social relationship adjustments in line with daily care requirements. In addition, people with ostomies may experience fatigue, pain, ostomy fecal leakage, peristomal skin complications, nausea, diarrhea, and constipation.^5^, ^6^

Specialized nurses play a significant role in improving the impacts of these problems and helping patients with ostomies return to their normal lives.^7^ An international declaration of the rights of patients with ostomies states that ostomy patients need to receive specialized pre‐ and postsurgical nursing care, either in hospitals or in the community.^8^ According to Sasaki et al.,^9^ patients with ostomies need specific qualified nursing care and multiprofessional follow‐up to meet their needs. Such care should be introduced in the preoperative period and continue throughout the period of recuperation and beyond. Furthermore, research has found that patients receiving care in hospitals with more and better trained staff have a superior postoperative outcome. In contrast, as reported by the Institute of Medicine, poor staffing and working conditions of registered nurses can threaten patient safety.^10^ Therefore, in both hospital and outpatient settings, medical and surgical nurses play an important role in assisting patients with ostomies to self‐manage disease‐related effects.^5^

Wound ostomy continence nurses (WOCNs) have an important role in the preoperative period. For example, it has been reported that patients whose ostomy sites are placed by a WOCN suffer fewer postoperative complications than those whose ostomy sites are placed by others.^4^ While WOCNs offer education and care to all patients with ostomies, they must rely on general nursing staff to deliver routine ostomy care.^11^ Having nursing staff who are knowledgeable and confident with providing ostomy care can significantly improve patient satisfaction with the overall care received.^12^ Patients undergoing ostomy surgery may express fears and ostomy‐related concerns; thus, all healthcare team members, including the WOCN, should be available to provide assistance and support to patients.^11^, ^13^ In addition, all acute care nurses should have the basic competence to provide postoperative care and educational support to new ostomy patients. This should include information on proper stoma assessment, the pouch system fitting, emptying and changing pouches, access to supplies, and basic stoma‐related problem‐solving skills.^11^

Providing care and preparing patients to live with an ostomy can be difficult for healthcare professionals. Academic courses generally do not emphasize physical stoma care, and as a result, healthcare professionals’ preparation to deal with changes in the lifestyles of patients with ostomies is not considered adequate.^8^ While there have been several studies on the quality of life of patients with ostomies, challenges related to living with a stoma, and associated complications after ostomy surgery,^14^ few studies have explored the knowledge, skills, and attitudes of nursing staff in relation to ostomy care.

Experience and education are two nursing characteristics that are highly associated with patient outcomes.^15^ There is mounting evidence of a correlation between adverse events and nurses’ levels of education and years of nursing experience.^10^, ^13^, ^16^ A recent systematic review by Audet et al.^16^ found that better nursing education and longer nursing experience were associated with benefits to patients in terms of lower risk of mortality and adverse events. Nurses’ knowledge of the needs of patients with ostomies and the implementation of appropriate ostomy care can prevent ostomy‐associated problems for patients following stoma surgery.^3^, ^7^ Moreover, the quality of life for patients with ostomies could be improved through the application of evidence‐based clinical nursing interventions.^3^, ^7^

In the Kingdom of Saudi Arabia, national healthcare has become a priority in the last decade, which has coincided with an influx of expatriate workers and the construction of modern hospitals. As a result, the majority of nursing staff in Saudi Arabia are not Saudi nationals, and all hospitals follow Western‐style care models.^17^ However, according to Murray et al.,^17^ this can contribute to difficulties caring for patients with ostomies from a Muslim background. The researchers suggested that this may be mitigated by having Saudi staff nurses who demonstrate specific aspects of cultural care to assist non‐Saudi staff nurses to adapt to the prevalent cultural differences.^17^

However, few studies have investigated the influence of nurses’ education and experience on ostomy care in Saudi Arabia. The purpose of this study was to determine the confidence of staff nurses in Saudi Arabia in providing ostomy care with regard to their knowledge, skills, and barriers to ostomy care. Second, we explored the link between nurses’ perceived confidence in ostomy care knowledge or skill and factors like nursing education, years of experience, and receiving ostomy care training in nursing school. This is important to study based on the high incidence of colorectal cancer in Saudi Arabia, which means many patients undergo ostomy surgery.

## 2 METHODS

### 2.1 Study design and population

This descriptive study used a convenience sample of nurses (*N* = 214) working in five specialized hospitals in Riyadh, Saudi Arabia. Collectively, these healthcare facilities have a total hospital bed number of between 600 and 1586. At the time of the study, only one of the hospitals had a dedicated certified stoma care nurse, while the others had a general nurse who undertook the role of a stoma care nurse. The study procedures were reviewed and approved by the human research ethics committee (approval number 21983) and the appropriate ethics review committee of each participating healthcare facility. The target population was any registered nurse working in the selected healthcare institutions. Nurses were made aware of the study via advertisements and information sessions. Information sessions were held in the hospitals’ nurses’ lounge at the time of shift handover. The nurses were informed about the study and how they could participate. Nurses who chose to participate were asked to sign a consent form and complete a questionnaire (see Section 2.2). The completion of the questionnaire required approximately 15–20 min.

### 2.2 Instruments

The questionnaire used was developed by Cross et al.^12^ It included questions related to demographic data, specialized ostomy nurse availability and referral services, ostomy care training in preservice education, frequency of ostomy care, and confidence in ostomy care on a 6‐point Likert Scale (1 = Strongly Disagree, 6 = Strongly Agree). The first section of the questionnaire focused on the nurses’ perceived confidence in ostomy care, while the second section investigated potential barriers to ostomy care. Confidence level items included questions relating to nursing staff confidence, skills, and supplies required for ostomy care. There were seven items to measure confidence in relation to knowledge and six in relation to skills. Questionnaire items relating to barriers were grouped into two areas, namely, beliefs (five questions) and work conditions (four questions). The questionnaire has been previously tested for validity and reliability by a panel of WOCNs.^11^

### 2.3 Statistical analysis

Numeric variables are summarized using descriptive statistical analysis. For relevant categorical variables, the frequency distribution, count, and percentage are reported. For continuous variables, descriptive statistics such as the mean and SD are reported.

Confidence levels in knowledge and skills for providing nursing stoma care were calculated by summing responses over a group of related question items. Factors that affect the level of confidence and perception of barriers were evaluated using Spearman's rank correlation coefficient. Alternative methods of statistics assuming unequal variances were administered. Linear regressions were used for factors with ordinal scales, including the number of years of nursing experience, frequency of ostomy care, and understanding of how to obtain and use ostomy supplies. The level of statistical significance was set at *p* < .05.

## 3 RESULTS

### 3.1 Demographics and work characteristics

The study sample consisted of 214 survey participants. The demographic profiles of the participants are summarized in Table 1. Most of the nurses surveyed worked with adults (*n* = 197, 95.1%), and a vast majority had a surgical focus (*n* = 176, 82.2%). More than half the nurses were nurse technicians (holding a nursing diploma; *n* = 136, 63.6%). The majority had received their nursing qualification in the Philippines (*n* = 140, 65.4%), followed by India (*n* = 41, 19.2%) and Saudi Arabia (*n* = 23, 10.7%). Approximately half of the nurses had 6–10 years of nursing experience (*n* = 106, 49.5%). A majority of the nurses were females (*n* = 159, 74.3%) with the dominant age group ranging from 30 to 39 years (*n* = 129, 60.3%).

**Table 1:** Participants demographics

Demographic characteristics	% (*N*)
Primary area of practice	
Adult	95.1% (197)
Pediatric	0.9% (2)
Both	7.0% (15)
Are you an	
Nurse specialist	36.4% (78)
Nurse technician	63.6% (136)
Primary area of focus	
Medical	12.1% (26)
Surgical	82.2% (176)
Critical Care	0.9% (2)
Rehabilitation	0.5% (1)
Other	4.2% (9)
Gender	
Male	25.7% (55)
Female	74.3% (159)
Age	
20–29	19.2% (41)
30–39	60.3% (129)
40–49	17.3% (37)
50–60	3.3% (7)
Country of training	
Philippines	65.4% (140)
Saudi	10.7% (23)
India	19.2% (41)
European	1.4% (3)
Other	3.3% (7)
Years of experience as a nurse (years)	
1–5	17.3% (37)
6–10	49.5% (106)
11–15	16.4% (35)
16–20	8.9% (19)
Above 20 years	7.9% (17)

Nurses were asked whether their facility had a certified ostomy nurse as part of the study's questions on work characteristics (see Table 2). Most answered “yes” (*n* = 154, 72.0%), despite only one hospital (*N* = 36) having a certified ostomy care nurse at the time of the study. Furthermore, most of the nurses answered that they had consulted a certified ostomy nurse in their facility (*n* = 184, 86.0%). The reasons for consulting ostomy nurses included dealing with a patient with a new ostomy (*n* = 73, 34.1%), ostomy complications (*n* = 57, 26.6%), and miscellaneous or other reasons (*n* = 80, 37.4%).

**Table 2:** Participants work characteristics

Work characteristics	% (*N*)
Is there a certified ostomy nurse at your facility	
Yes	72% (154)
No	21.5% (46)
I don't know	6.5% (14)
Training regarding ostomy care in nursing school	
Yes	57% (122)
No	34.1% (73)
I don't remember	8.9% (19)
Consult certified ostomy nurse in my facility/practice when I have a patient with a new ostomy	
Yes	86% (185)
No	9.3% (20)
There is no ostomy nurse	4.2% (9)
Other reasons you would consult a certified ostomy nurse	
Dealing with a new patient for education	34.1% (73)
Ostomy complications	26.6% (57)
Supplies	1.9% (4)
Miscellaneous or other reasons	37.4% (80)
Frequency of delivering ostomy care	
Never	4.2% (9)
Rarely (once or twice a year)	29.0% (62)
Sometimes (every month or two)	16.8% (36)
Frequently (a couple of times a month)	20.6% (44)
Always (on a weekly or daily basis)	29.4% (63)

More than half the nurses answered that they had received training in ostomy care in nursing school (*n* = 122, 57.0%). Most indicated they had provided ostomy care at some time (*n* = 205, 95.8%), with only a few nurses having never provided ostomy care (*n* = 9, 4.2%). Half the surveyed nurses (*n* = 107, 50%) provided ostomy care regularly (“a couple of times a month,” *n* = 44, 20.6%; “on a weekly basis,” *n* = 63, 29.4%).

### 3.2 Perceived knowledge and skill confidence

Table 3 shows the mean scores and SDs for the nurses’ knowledge and skill in different areas of ostomy and stoma care. In terms of knowledge, the nurses demonstrated high confidence in preventing skin problems, with a mean rating of 5.02 (SD = 0.781); most nurses either “agreed” (*n* = 120, 56%) or “strongly agreed” (*n* = 54, 25%) to being confident at preventing skin problems. The nurses were somewhat less confident in dealing with peristomal skin problems (mean = 4.78, SD = 0.956), with just 48% (*n* = 103) indicating “agree” and 21% (*n* = 45) indicating “strongly agree.”

**Table 3:** Participants perceived knowledge and skills

Knowledge and skills statement	Mean	SD
Knowledge statement: I feel confident		
Knowing the difference between colostomy, ileostomy and urostomy	4.94	0.930
Prevention of skin problems	5.02	0.781
Dealing with the odor	4.91	0.872
When to attach to a larger drainage bag if needed	4.78	1.005
Ordering the correct supplies	4.86	0.956
Dealing with peristomal skin problems	4.77	0.956
Knowledge of nutrition for patients with ostomies	4.79	0.935
Skill statement: I feel confident		
Emptying an ostomy appliance	5.14	0.841
Changing an ostomy appliance	5.00	0.906
Cutting the wafer the appropriate size	5.00	0.991
Measuring the stoma	4.89	1.054
Applying the paste, if needed	4.86	1.051
How to attach to a larger drainage bag, if needed	4.78	1.010

The nurses were also asked about their confidence in terms of skills for ostomy care. Most thought they could adequately empty an ostomy appliance (mean = 5.14, SD = 0.841), which received the highest mean score for this section; 48% (*n* = 103) of nurses “agreed” and 36% (*n* = 77) “strongly agreed” that they felt confident when emptying an ostomy appliance. The lowest mean score for skills was for the ability to attach a larger drainage bag (mean = 4.78, SD = 1.010), with only 43% (*n* = 94) of nurses nominating “agree” and 24% (*n* = 52) nominating “strongly agree.”

### 3.3 Belief statements and work conditions

The responses to the “beliefs” statements (Table 4) indicated that most nurses believed patients should care for their own ostomy, if they are so able (mean = 5.03, SD = 0.973, 42% “agree” (*n* = 89), 36% “strongly agree” (*n* = 76)). Nevertheless, some nurses identified that patients wanting to care for their ostomy themselves would refuse ostomy care by nurses (mean = 3.56, SD = 1.323, 3% “agree” (*n* = 7), 25% “strongly agree” (*n* = 54)). Likewise, several of the nurses were concerned about doing something incorrectly (mean = 3.44, SD = 1.351, 5% “agree” (*n* = 5), 25% “strongly agree” (*n* = 54)) or harming the patient's stoma (mean = 3.31, SD = 1.430, 2% “agree” (*n* = 5), 24% “strongly agree” (*n* = 53)).

**Table 4:** Belief statements and work conditions

Belief and work condition statement	Mean	SD
Belief statements		
Ostomy care is unpleasant, making me at times reluctant to do it	3.11	1.352
I am concerned I may do something incorrectly	3.44	1.351
I am concerned I may harm my patient's stoma	3.31	1.430
Patients refuse ostomy care, wanting to care for their ostomy themselves	3.56	1.323
Patients should care for their own ostomy, if they are able	5.03	0.973
Work conditions		
Ostomy supplies are easy to order in my facility	4.49	0.938
Lack of time is a factor in delivering ostomy care promptly	3.74	1.362
Lack of time is a factor in delivering ostomy care properly	3.70	1.468
Ostomy patient teaching materials are available in my facility	4.47	1.201

The data pertaining to work conditions demonstrated that the majority of nurses found ostomy supplies easy to order in their facility (mean = 4.49, SD = 0.938), with good availability of ostomy patient teaching materials (mean = 4.47, SD = 1.201). However, the nurses also reported lack of time as a factor in delivering ostomy care promptly (mean = 3.74, SD = 1.362) and properly (mean = 3.70, SD = 1.468).

### 3.4 Correlation between nurses’ perceived confidence level and experience, education, and training

There was a significant positive correlation (*p* < .001) between nurses’ years’ experience and perception of their ostomy care knowledge, with confidence increasing as experience grew (Table 5). Based on this, 48.65%  of nurses with 1–5 years of experience selected “agree” or “strongly agree” to all of the survey items relating to their confidence in knowledge for ostomy care. Of nurses with more experience, 70.75% (6–10 years’ experience), 94.29% (11–15 years’ experience), 73.68% (16–20 years’ experience), and 100% (>20 years’ experience) nominated “agree” or “strongly agree” for their knowledge confidence level. As a result, it can be concluded that nurses with more years of experience perceive themselves to be more knowledgeable about stoma care.

**Table 5:** Correlation between nurse perceived confidence level in knowledge for ostomy care and years of experience

Symmetric measures years of experience * knowledge					
		Value	Asymptotic standard error	Approximate *T*	Approximate significance
Ordinal by Ordinal	Spearman correlation	0.338	.062	5.223	.000*
*N* of valid cases		214			

Years of experience * knowledge cross‐tabulation							
		Disagree	Slightly disagree	Slightly agree	Agree	Strongly agree	Total
How many years have you been a nurse?	1–5 years	1	2	16	13	5	37
	6–10 years	0	6	25	61	14	106
	11–15 years	0	0	2	19	14	35
	16–20 years	0	0	5	7	7	19
	Above 20 Years	0	0	0	12	5	17
Total		1	8	48	112	45	214

* Correlation is significant at the .05 level (two‐tailed).

A significant positive correlation (*p* = .00) was also identified between the nurses’ years of experience and their confidence in providing ostomy care (see Table 6). The results revealed that 51.35% of nurses with 1–5 years of experience “agreed” or “strongly agreed” to being confident in their ostomy care skills. In addition, 77.36% of nurses with 6–10 years of experience nominated “agree” or “strongly agree” for their skill confidence level, while 91.43% of nurses with 11–15 years’ experience and 84.21% of nurses with 16–20 years’ experience indicated “agree” or “strongly agree.” Notably, 100% of nurses with more than 20 years’ experience nominated “agree” or “strongly agree” for their skill confidence level. These findings demonstrate that nurses with more than 20 years of experience have more confidence in their ostomy care skills than nurses with fewer years of experience.

**Table 6:** Correlation between nurse perceived confidence in skills for ostomy care and years of experience

Symmetric measures of years of experience * skills					
		Value	Asymptotic standard error	Approximate *T*	Approximate significance
Ordinal by ordinal	Spearman correlation	0.248	.068	3.732	.000
*N* of valid cases		214			

Years of experience * skills cross‐tabulation							
		Disagree	Slightly disagree	Slightly agree	Agree	Strongly agree	Total
How many years have you been a nurse?	1–5 years	1	3	14	8	11	37
	6–10 years	1	6	17	62	20	106
	11–15 years	0	0	3	15	17	35
	16–20 years	0	0	3	10	6	19
	Above 20 years	0	0	0	12	5	17
Total		2	9	37	107	59	214

* Correlation is significant at the .05 level (two‐tailed).

There was a significant correlation between receiving ostomy care training in nursing school and knowledge confidence level (*p* < .001). For nurses who received ostomy care training in nursing school (*N* = 122), 80.33% “agreed” or “strongly agreed” with having confidence in their ostomy care knowledge. In contrast, only 58.49% of nurses who did not receive ostomy care training in nursing school answered “agree” or “strongly agree” to having confidence in their ostomy care knowledge (see Table 7). These findings suggest that nurses who receive ostomy care training in nursing school have more confidence in their ostomy care knowledge than nurses who do not receive (or do not recall receiving) ostomy care training in nursing school.

**Table 7:** Correlation between nurse perceived confidence in knowledge of ostomy care and training received on ostomy care in nursing school

Symmetric measures received training ostomy care in nursing school * knowledge					
		Value	Asymptotic standard error	Approximate *T*	Approximate significance
Ordinal by ordinal	Spearman correlation	−0.228	.067	−3.385	.001
*N* of Valid Cases		210			

Received training ostomy care in nursing school * knowledge cross‐tabulation							
I received training regarding ostomy care in nursing school?	Yes	1	2	21	66	36	126
	No	0	4	19	38	12	73
	I don't remember	0	2	7	5	1	15
Total		1	8	47	109	49	214

* Correlation is significant at the .05 level (two‐tailed).

There was also a significant correlation between nurses’ confidence in their ostomy care skills and receiving ostomy care training in nursing school (*p* = .000; see Table 8), with 84.43% and 73.97% of nurses who did and did not receive ostomy care training in nursing school, respectively, nominating “agree” or “strongly agree” to having confidence in their ostomy care skills. As a result, nurses who receive ostomy care training in nursing school appear to have more confidence in their ability to provide ostomy care than nurses who do not receive (or do not recall receiving) ostomy care training.

**Table 8:** Correlation between nurse perceived confidence in skill of ostomy care and training received on ostomy care in nursing school

Symmetric measures received training ostomy care in nursing school * skill					
		Value	Asymptotic standard error	Approximate *T*	Approximate significance
Ordinal by ordinal	Spearman correlation	−0.260	.066	−3.889	.000
*N* of valid cases		210			

Received training ostomy care in nursing school * skills cross‐tabulation							
I received training regarding ostomy care in nursing school?	Yes	1	2	16	61	46	126
	No	1	4	14	39	15	73
	I don't remember	0	3	6	5	1	15
Total		2	9	36	105	62	214

* Correlation is significant at the .05 level (two‐tailed).

There was no statistically significant relationship between nurses’ level of education and their perceived confidence in their ostomy care knowledge or skills (*p* > .05).

## 4 DISCUSSION

The purpose of this study was to investigate nursing staff's perceived level of confidence in their ostomy care knowledge and skills, as well as the barriers they face when caring for patients with stomas. In addition, we determined the correlation between a nurse's confidence in their ostomy care knowledge and skills and their nursing degree, years of nursing experience, and receiving ostomy care training in nursing school. In a study on patient satisfaction during a hospital stay, it was found that many patients are not entirely satisfied with the quality of care they receive.^12^ Our clinical experience in caring for people with ostomies suggests that individuals with ostomies do not have all their needs met when receiving care in healthcare institutions. This may be caused by nurses’ lack of confidence and knowledge in caring for individuals with an ostomy.

One of the main findings of this study was the existence of significant differences in nurses’ confidence levels for providing ostomy care based on their knowledge, skills, and barriers. Our survey participants reported having similar confidence in their ostomy care knowledge and skills to those in the study by Cross et al.^12^ Nurses who are knowledgeable, skillful, and comfortable providing care could improve the quality of life and satisfaction of patients and their families in hospital settings.^18^ In contrast, healthcare professionals who lack confidence in providing care decrease the quality of care for hospitalized patients.^19^

This study also explored the potential barriers that may limit nurses from delivering ostomy care. The responses to the “working conditions” statements showed that nurses found ostomy supplies easy to order in their facility. This also indicates that the nurses had a good understanding of the products required for ostomy care. Contrary to the findings of this study, Cross et al.^12^ found slight agreement on the simplicity of ordering supplies between nurses. This difference may be explained by the variance in the population of the studies and the supply order systems used. All nurses have an important role in health education to support and encourage patients to perform self‐care.^11^

The survey results showed that nurses with more years of nursing experience had higher confidence in their ostomy care knowledge and skills. This finding is in line with a previous study that found a link between nursing competence and clinical experience.^20^ Similarly, Cross et al.^12^ found a significant correlation between number of years’ nursing experience and confidence in ostomy care knowledge and skills. Furthermore, Hibbert^21^ reported that the frequency of involvement in ostomy care affects the confidence levels of nurses. In contrast, Bhzeh et al.^13^ proposed that more working experience has no impact on knowledge in ostomy care; nevertheless, this may be because the nurses involved in the study lacked experience in providing ostomy care. Another study found that nurses in China with more than 26 years of experience and working in high practice positions had significantly lower scores in knowledge and skill of ostomy care than nurses in bedside care or those with 15–25 years of experience. This finding may be because senior nursing staff with many years’ experience are often employed in management positions; thus, they have less direct contact with patients’ ostomy care.^22^

This study has documented that nurses gain more confidence in their knowledge and skill as they gain more years of clinical experience. The role of nurses is critical in determining how to educate patients to develop their knowledge and skills in relation to ostomy care. This may also be a factor in areas such as ostomy management and self‐care, which affect patients’ adjustment to life with an ostomy.^8^ The role of experience, as established in this study, affirms its involvement in improving the capabilities and confidence of nurses who care for patients with ostomies.^12^, ^20^, ^21^

Training is critical to nurses’ capacities to deliver ostomy care.^12^ This conforms to the generally accepted rule that possessing appropriate knowledge enhances confidence in practice.^12^ According to the findings of this study, receiving ostomy care training in nursing school was associated with a high level of perceived confidence in knowledge and skill in providing ostomy care. Ostomy training helps to improve knowledge of different aspects of ostomy care, which contributes to confidence, as the nurses are sure of their capability to conduct care.^12^, ^13^ The findings showed a correlation between nurses who received ostomy care training in nursing school and perceived knowledge confidence level, which is consistent with other studies.^6^ However, nurses who had no specialized training had inadequate and significantly worse knowledge than nurses who had completed ostomy care training.^6^, ^13^ Li et al.^22^ evaluated staff nurses who were practicing and training to care for patients with intestinal ostomies, and noted that first‐line nursing staff had little educational training in ostomy care and less than optimal confidence in delivering ostomy care. These findings highlight the importance of specialized training for nurses to improve their ability and confidence in delivering stoma care.

In terms of education, there has been a reported benefit of nursing education at the bachelor's level or higher, including a strong association with fewer adverse outcomes for patients.^10^ A previous study indicated that nurses’ education level has a significant impact on their knowledge and competency for providing ostomy care.^13^ However, our findings did not show a correlation between education and confidence in ostomy care knowledge or skill. This corresponds to the findings of a study by Friese et al.,^10^ which found that nursing staff *without* university qualifications had higher competence scores in patients education than their more educated counterparts.

## 5 CONCLUSIONS

This study is one of the first to investigate how nurses in Saudi Arabia provide ostomy care. Improvements in the quality of ostomy care provided by nurses could reduce the number of complications for patients with ostomies. The findings revealed that nurses’ perceived knowledge and skills in caring for patients with ostomies were lacking, particularly when they had fewer years’ experience or had not received ostomy care training in nursing school. There is a need for nurses to improve their ostomy management knowledge and skills for clinical practice. With regard to nurses’ beliefs about ostomy care, the participants believed that independence was the ultimate aim, with patients caring for their own ostomies; however, it is important to balance this with supportive care as the patient adjusts. Nurses must have opportunities and incentives to upgrade their status pertaining to becoming specialized nurses’ in caring for patients with ostomies while also increasing patients’ access to such specialized nurses in daily practice. There is evidence to suggest that ostomy care training in nursing school and years of nursing experience are associated with the level of confidence in knowledge and skill in providing ostomy care to patients. More research is needed to better understand the possible links between nursing staff education and experience with ostomy care, as well as the benefits to patients and organizations.

## CONFLICT OF INTERESTS

The authors declare that there are no conflict of interests.

## AUTHOR CONTRIBUTIONS

The authors confirm contribution to the paper as follows: *conception and design of the work*, *data analysis and interpretation*, and *drafting the article*: Aishah Alenezi; *critical revision of the article*: Aishah Alenezi, Amanda Kimpton, Ian McGrath, and Karen Livesay; and *final approval of the version to be published*: Aishah Alenezi, Ian McGrath, Amanda Kimpton, and Karen Livesay.

## Data Availability

The data that support the findings of this study are available from the corresponding author upon reasonable request.
